# The Vaginal Virome in Women’s Health and Disease

**DOI:** 10.3390/microorganisms13020431

**Published:** 2025-02-16

**Authors:** Kyra l’Cess Orton, Cynthia L. Monaco

**Affiliations:** 1 Department of Microbiology and Immunology, University of Rochester Medical School, Rochester, NY 14642, USA; 2 College of Human Medicine, Michigan State University, East Lansing, MI 48824, USA; 3 Division of Infectious Diseases, Department of Medicine, University of Rochester Medical School, Rochester, NY 14642, USA

**Keywords:** vaginal virome, female reproductive tract, bacteriome, vaginal microbiota transplant, bacterial vaginosis

## Abstract

Most research on the vaginal microbiome has focused on bacterial communities (the bacteriome), but viruses, including eukaryotic viruses and bacteriophages, are also important players in vaginal health and disease states. In this review, we will briefly discuss the bacterial microbiome, delve into what is known about the vaginal virome and its impact on women’s health, and finish with novel vaginal microbial or microbial-derived therapeutics on the horizon. More studies on the vaginal virome and its impact on women’s health are needed to better prevent and treat gynecological, reproductive, and neonatal diseases.

## 1. Introduction

The vaginal mucosa, similar to other mucosal sites, is the first barrier of protection against invading pathogens. The vaginal microbiome is an important component of this defense and carries out functions that help to protect against a number of urogenital diseases, including yeast infections, bacterial vaginosis, urinary tract infections, HIV, and other sexually transmitted infections [1]. The vaginal microflora, in turn, are impacted by host factors including gestational status, menstrual cycle, sexual activity, age, contraceptive use, diet, lifestyle factors such as tobacco use, immune status, race/ethnicity, and environment [1,2,3,4]. The vaginal microbiome is composed of a variety of bacteria, yeast, viruses, and protozoa that play a role in women’s health, where disruptions in a woman’s normal vaginal microbiome contribute to vaginal disease states through transkingdom interactions, including with the host mucosa and immune system. In this review, we will dive deeper into understanding the relationship between the vaginal microbiome, in particular the vaginal viral microbiome (virome), and disease, as well as discuss new and emerging microbiome-targeted treatments for these disease states.

In the last three years, there has been a doubling of the manuscripts detailing the vaginal virome and its impact on women’s health using next-generation sequencing [5,6,7,8,9,10,11,12,13,14] compared to the decade prior [15,16,17,18,19]. The last few years have also seen the development of promising new microbial-based therapies, highlighting the growing importance of a better understanding of all members of the microbiota. The present review summarizes knowledge on the vaginal virome’s composition, interaction with other microbes in its environment, and its contribution to women’s health. We searched for and included all research manuscripts published in English within the last 10 years and indexed within the PubMed database that detail the vaginal virome through next-generation sequencing using mesh terms including “vaginal virome”, “female genital tract virome”, and “vaginal virus”. For transkingdom interactions and microbial-based therapeutics summation, we searched all types of papers, including reviews, retrospective analyses, and research studies, within the last decade, prioritizing the last 5 years and using mesh terms such as “vaginal microbiome”, “current treatments for bacterial vaginosis”, and “vaginal microbiome transplantation”.

## 2. The Vaginal Bacteriome

The vaginal bacterial microbiome (bacteriome) is the best-studied component of the vaginal microbiome and is generally divided into lower-diversity *Lactobacillus*-dominant (LD) bacteriomes versus higher-diversity non-LD (NLD) bacteriomes dominated by facultative and obligate anaerobic flora. These distinctions have clinical and biological relevance. LD bacteriomes tend to show lower levels of localized inflammation, whereas higher diversity NLD bacteriomes are associated with vaginal disease states, including BV, preterm birth, infertility, higher transmission and acquisition of sexually transmitted infections (STIs) including HPV and HIV, and increased risk of cervical cancer [2,3,4,15,20,21]. The most prominent *Lactobacillus* species present in the vaginal bacteriome include *L. crispatus*, *L. gasseri*, *L. jensenii*, and *L. iners* [1,3]. Lactobacilli are known producers of lactic acid, hydrogen peroxide, and other antimicrobial and antifungal products that prevent the overgrowth of pathogenic bacteria and fungi [3,22].

### 2.1. Bacteriome Communities

**Figure 1:**
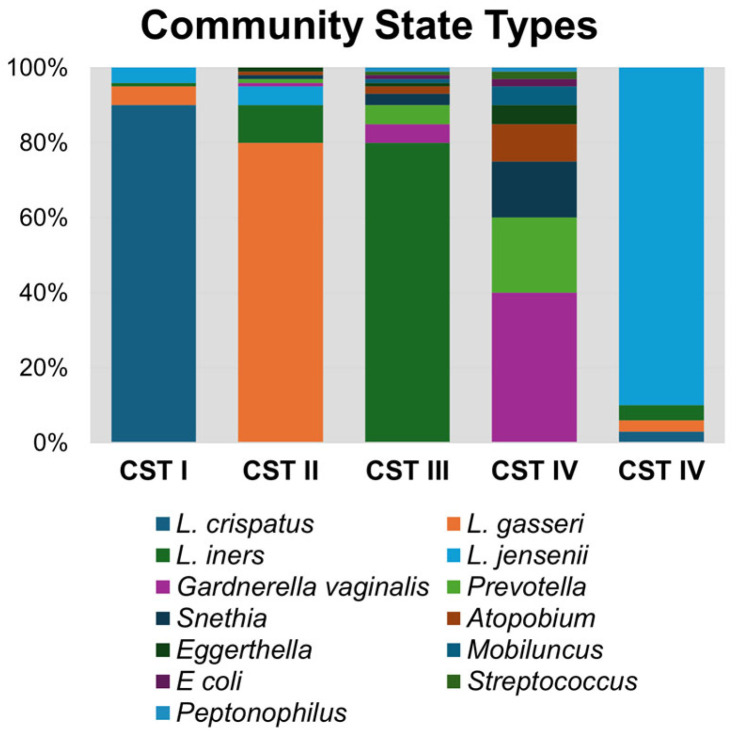
Community state type (CST) bacterial taxa composition. A representative synthetic bacteriome is graphed illustrating relative abundance of bacterial genera within each CST group.

Unlike in other biological niches, it is well established that vaginal bacteria form defined community groupings that are similar across populations. The most widely used classification method for vaginal bacteriomes is known as community state types (CSTs; Figure 1), which are numbered according to the dominant species of that group. CST I, II, III, IV, and V correspond with bacteriome domination by *L. crispatus*, *L. gasseri*, *L. iners*, anaerobic bacteria, and *L. jensenii*, respectively [3]. CST IV is notable for having diverse anaerobic bacteria, including *Gardnerella vaginalis*, *Prevotella bivia*, *Sneathia sanguinis*, *Mobiluncus mulieris*, and *Eggerthella lenta*, among others [3,23,24]. Lactobacilli, in particular *L. crispatus*, are known to reduce the response and activity of inflammatory compounds, whereas microbial communities with a predominance of *Gardnerella* or other anaerobes are known to produce pro-inflammatory cytokines, such as IL-6, IL-8, and TNF-α [15]. Bacteriomes dominated by anaerobic bacteria (CST IV) or *L. iners* (CST III) are associated with higher rates of reproductive tract inflammation and pathologies, such as bacterial vaginosis (BV) and HIV acquisition and transmission [1,3,22]. On the other hand, CST I, II, and V are associated with decreased vaginal inflammation and improved health outcomes and are protective against urogenital diseases.

Baseline vaginal bacteriome composition differs by geographic area, ethnicity, and race. About 90% of Caucasian women in North America have LD bacteriome communities, while only 60–65% of Black women in the US and 37–62% of Black women in Sub-Saharan Africa have LD bacteriomes. Black and Hispanic women are more likely to have higher diversity vaginal bacteriomes reflective of CST III and IV, hence the prevalence of conditions such as BV is significantly greater in Black and Hispanic women, at rates of 51.6% and 32.1% respectively, compared to Caucasian women, who have a prevalence rate of 23.2% [3,25].

### 2.2. Impact of the Bacteriome on HIV Acquisition and Pathogenesis

High diversity bacteriomes are associated with an increased rate of transmission and acquisition of STIs, including HIV. In 2023, 1.3 million people contracted HIV globally, with Sub-Saharan Africa disproportionately affected, including women and girls who accounted for 62% of all new HIV infections in this region [26]. Baseline vaginal bacteriomes contribute to the high rate of HIV acquisition in this population. In a prospective study to ascertain risk factors for HIV acquisition, vaginal swab samples were collected and analyzed from 236 HIV-negative women at risk for HIV acquisition [15]. Women with *L. crispatus*-dominated vaginal bacteriomes did not acquire HIV during their observation period. On the other hand, women with intermediate diversity *L. iners*-dominant vaginal communities and NLD bacteriomes acquired HIV at greatly increased rates compared to *L. crispatus*-dominated vaginal bacteriomes. The authors concluded that non-*iners* LD vaginal bacterial communities were protective against HIV acquisition and other pathogenic microorganisms, and those with *L. iners*-dominated communities and high diversity NLD bacteriomes were more susceptible to contracting HIV [15]. The mechanism behind this is two-fold. Inflammatory cytokines produced by *L. iners*-dominant and NLD bacteriomes cause not only increased permeability of the vaginal epithelium and cervical cell layers to HIV but also recruit CD4+ HIV target cells to the vaginal mucosa [15,27]. The underlying difference in mucosal permeability relies on the levels of lactic acid (LA) isomers. Cervicovaginal mucus with high levels of D-LA isomer produced by *L. crispatus* yields a low pH, which promotes carboxyl group hydrogen bonding between HIV virion glycans and surface mucins. This bonding causes HIV virion immobilization and prevention of HIV invasion into the genital mucosa [28]. Therefore *L. crispatus*-dominant bacteriomes have multiple mechanisms to protect against HIV infection, demonstrating the complex interplay between the vaginal bacteriome, host, and viral populations.

### 2.3. Postmenopausal Bacteriome

The decline in estrogen levels after menopause significantly impacts the vaginal microbiota, usually resulting in a reduced quantity of lactobacilli and other lactic acid-producing bacteria [29,30]. There is a transition to fewer LD bacteriomes and increased bacteriome diversity (CST IV) in the postmenopausal period, including increased abundance of *Prevotella*, *Streptococcus*, *Gardnerella*, and *Anaerococcus* [29,30,31]. CST IV, linked with poor reproductive health outcomes, has also been linked with a greater incidence of vaginal atrophy and dryness after menopause [29]. No studies to date have investigated the vaginal virome of postmenopausal women, but we would expect the virome to follow a similar trend as the bacteriome with increased diversity during the postmenopausal period based on the virome studies of BV detailed below [5].

## 3. The Vaginal Virome

The vaginal virome is comprised of eukaryotic viruses that infect human cells and bacteriophages, which infect the bacteriome. The majority of vaginal virome studies have focused on eukaryotic DNA viruses, such as *Papillomaviridae*, *Herpesviridae*, *Anelloviridae*, *Polyomaviridae*, *Poxviridae*, and *Adenoviridae*, through PCR-based techniques [32]. However, eukaryotic viruses usually comprise less than 5% of viral sequences found in the human vagina. More recent studies have attempted unbiased surveys of vaginal viruses (see Table 1), though RNA viruses are still understudied.

**Figure 2:**
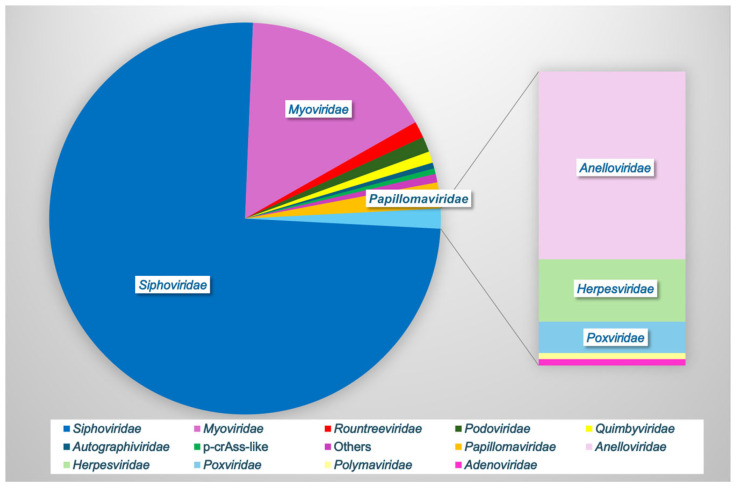
Vaginal viral family relative abundance. Bacteriophages comprise the majority of the vaginal virome (*Siphoviridae*, *Myoviridae*, *Rountreeviridae*, *Podoviridae*, *Quimbyviridae*, *Autographiviridae*, and p-crAss-like phage), while eukaryotic viruses (*Papillomaviridae*, *Anelloviridae*, *Herpesviridae*, *Poxviridae*, *Polyomaviridae*, and *Adenoviridae*) comprise a small minority. Data based on [5,13].

In one of the largest analyses to date, Huang et al., 2024, combined vaginal metagenomic sequencing data from 32 studies to compile a reference database of over 33,000 microbial genomes, including 4263 species-level viral operational taxonomic units (vOTUs) [13]. Rarefaction showed that these viral sequences did not reach a plateau, suggesting that other undiscovered viruses may be present in the vaginal vault [13]. Furthermore, 85.5% of the vOTUs were not found in other virome databases, indicating the significant unexplored viral diversity in this unique niche [13]. Only 66% of the vOTUs could be assigned to a known viral family, with the most frequently observed families being *Siphoviridae* (76% of known families), *Myoviridae* (16%), *Papillomaviridae* (2%), *Rountreeviridae* (1.4%), *Podoviridae* (1.3%), *Quimbyviridae* (<1%), *Autographiviridae*, crAss-like, and others (Figure 2). Apart from *Papillomaviridae*, the other top families (98%) were all bacteriophages [13]. Only 70 of the 4263 vOTUs were assigned to eukaryotic viruses, highlighting the importance of bacteriophages within this environment.

### 3.1. Vaginal Eukaryotic Viruses

Eukaryotic viral STIs have been well-studied through PCR-based techniques and are known contributors to vaginal disease [32]. The vaginal vault represents a physical and immunological barrier against the invasion of pathogenic organisms, often through the activation of pattern recognition receptors to trigger localized inflammation and viral clearance. Some DNA eukaryotic viruses, such as anelloviruses, are ubiquitous and considered commensals, but eukaryotic viral STIs, such as members of the *Polyomaviridae*, *Herpesviridae*, and *Poxviridae* families, are associated with increased vaginal inflammation, vaginal mucosa barrier degradation and adverse reproductive health and gynecological outcomes, including preterm birth, bacterial vaginosis (BV), and ulcerogenital disease [16,33,34,35,36,37,38,39]. Zika virus can be sexually transmitted and has been found in the female reproductive tract, which may have profound implications for vertical transmission in pregnant women and risk of birth defects [40]. However, it has not been as well-studied in women as in men, and the clinical relevance of vaginal shedding is unclear. Coinfection with multiple viruses can act synergistically to increase mucosal damage. HIV is associated with increased risk of urogenital herpes simplex virus (HSV) lesions and human papillomavirus (HPV) persistence [41,42,43], while other viral STIs can increase the risk of HIV transmission and shedding [44,45,46,47]. These examples illustrate the complex interplay of the vaginal eukaryotic virome in vaginal health.

### 3.2. The Vaginal Phageome

Bacteriophages are the most abundant vaginal viral group (Figure 2) and can directly interact with the host to elicit a humoral immune response. Bacteriophage DNA can also be recognized by cellular pattern recognition receptors, including toll-like receptor (TLR)-9, to induce an inflammatory type-1 interferon (IFN) response [48]. Bacteriophages can indirectly stimulate the host immune response through the regulation of bacterial populations and the release of bacterial products such as endotoxin during bacterial lysis [32]. Functional annotation of the vaginal virome shows about 20% of bacteriophage genes identified were viral auxiliary metabolic genes, including numerous peptidoglycan lyase/hydrolase-related genes, which may facilitate bacterial cell wall degradation during phage infection [13].

Bacteriophages may exhibit one of two different life cycles: lysogenic or lytic. Lytic bacteriophages enter and hijack the bacterial replication machinery to produce often thousands of progeny viruses, resulting in lysis and death of the host cell. Lysogenic bacteriophages, on the other hand, integrate into the host genome, replicating when the host replicates. Lysogenic bacteriophages can carry virulence factors that may provide survival advantages to the bacterial host, including conferring tolerance to ecological stressors, superinfection immunity, increased pathogenicity, and antibiotic resistance [49,50]. However, when the environment becomes unfavorable, lysogenic phages can rapidly switch life cycles and become lytic, thus subsuming bacterial functions and ultimately causing host bacterial death [51]. This life cycle switch can be triggered through bacteriophage quorum sensing mechanisms [51,52]. Prior work has shown an increased prevalence of lysogenic phages in women with BV, which may provide survival benefits to their hosts to improve persistence [5]. Ex vivo studies showed lysogeny is also common in *L. crispatus*, at rates up to 77% of clinical isolates [53]. Further, host ranges of clinical *Lactobacillus* phages were broad and included multiple *Lactobacillus* species, and because phages that are lysogenic in one species can be lytic in other species, this may be one way lactobacilli maintain the stability of the vaginal microbiome [54].

### 3.3. Transkingdom Dynamics of the Vaginal Microbiome

Bacteriome shifts from LD to non-LD can occur rapidly. While factors that trigger shifts, such as menstrual cycle and sexual activity, are known, the mechanisms and dynamics of these shifts (i.e., loss of *Lactobacillus* vs. overgrowth of other bacteria causing suppression of *Lactobacillus*) and relation to other factors (phage blooms, genetics, and environmental changes) are unclear. Commercially available yogurts containing *Lactobacillus* strains have inducible phages that can inhibit vaginal lactobacilli [55], suggesting that exogenous introduction may play a role in bacteriome shifts.

Recently, Hugerth et al. used metagenomic sequencing (MGS) to characterize daily vaginal swabs from 49 healthy reproductive-age women to assess the dynamics of the vaginal bacteriome and virome [11]. They found the bacterial communities fluctuated considerably in many individual women over time (6/49 varied with menstruation and 11/49 were unstable throughout the cycle). Women with stable non-*iners* LD bacteriomes exhibited 1 log higher levels of bacteriophage counts, with bacteriophage spikes noted with the bacteriome shifts. Higher levels of bacteriophage reads were also noted during menstruation in women whose bacteriome shifted during menstruation, suggesting that bacteriophages may contribute to the resolution of the dysbiosis in these women. While phages are believed to play a role in the stability of the vaginal bacteriome, even daily sampling was insufficient to ascertain if phage blooms precede and, hence, may cause bacteriome shifts [11]. Further studies would be needed to justify phage therapy for the treatment of BV or promoting LD bacteriomes for vaginal health.

### 3.4. Benefits and Limitations to Metagenomic Sequencing Studies

The majority of viruses are unculturable, making traditional methods to identify novel viruses impossible. Viruses also lack a universal sequencing marker, such as the bacterial 16S ribosomal RNA gene widely used for bacteriome research, negating the possibility of lower-cost sequence amplification techniques [56,57]. Over the last two decades, MGS has exponentially expanded the number of known viruses, creating a new field of study, the virome [58]. However, there are limitations and biases associated with virome MGS. MGS is expensive, which until recently has resulted in small sample sizes for most studies, limiting statistical power to determine associations with disease. MGS also has lower sensitivity than targeted PCR-based methods but is the only method currently available to identify unculturable, novel viruses [57]. RNA viruses in particular continue to be understudied due to decreased sequencing yield with combined DNA/RNA viral amplification techniques and poor identification in taxonomic pipelines due to divergent genomes [59].

Viruses overall represent a minute fraction of the total biomass of any sample, often requiring special techniques to amplify viral sequences sufficiently for detection by MGS [60]. Unfortunately, there are no standardized nucleic acid extraction protocols, amplification protocols, or taxonomic identification pipelines for virome MGS, and many techniques introduce bias [56,57]. For instance, nucleic acid extraction methods that involve filtration for viral sequence enrichment can filter out larger virions, including crAssphage. Commonly used amplification techniques to increase viral yield, such as rolling circle amplification, bias toward small circular DNA viral genomes [57]. Perhaps most importantly, viral identification is biased toward known viruses, as these are present in viral taxonomic databases. The majority of sequences in virome-sequencing datasets cannot be mapped to known viruses, and have been dubbed the “viral dark matter” [61]. Current bioinformatics tools have difficulty identifying highly divergent viruses, but deep artificial neural networks have been used to “learn” viral features and more successfully predict viral sequences with higher accuracy [61], including the recent identification of over 160,000 new putative RNA viruses using a deep learning algorithm [59]. While the field faces challenges, the evolving methods and bioinformatics tools will continue to ensure new findings for many decades to come.

## 4. The Contribution of the Vaginal Virome to Women’s Health

The following examples illustrate the well-documented role the vaginal virome plays in vaginal health and disease states (Table 2).

### 4.1. Cervical Cancer

**Figure 3:**
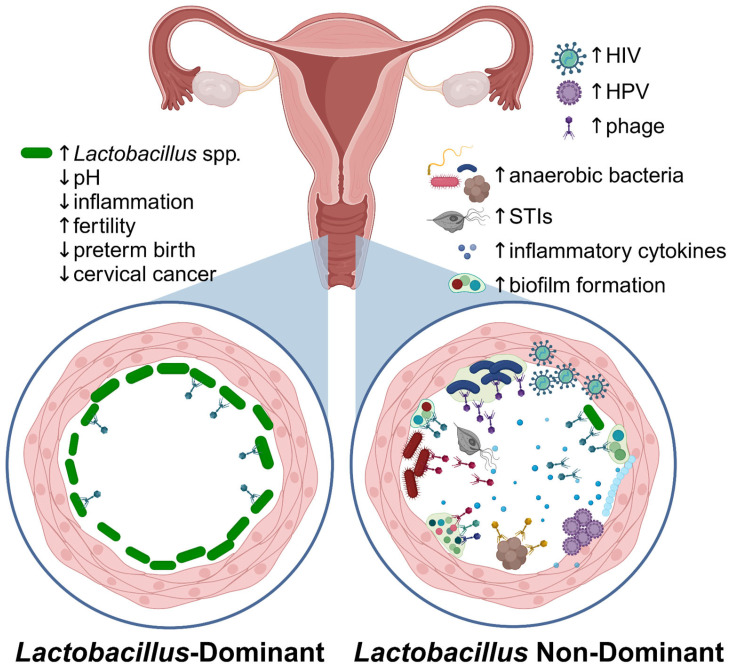
The vaginal microbiome in health and disease. Callouts illustrate differences in the microenvironment in Lactobacillus-dominant versus non-Lactobacillus-dominant (i.e., BV) vaginal bacteriomes. Figure created in Biorender.

HPV, the causative agent of cervical cancer, is the most prevalent vaginal eukaryotic virus worldwide, and the most common sexually transmitted virus [62]. HPV is a small DNA virus that infects squamous epithelial cells but is often cleared within 6-18 months after acquisition. However, persistent infection by certain HPV genotypes is associated with a high risk of progression to anogenital and oropharyngeal cancers (HR-HPV) or low risk of progression (LR-HPV), the latter of which cause anogenital warts [20]. The link between HPV and cervical cancer is better reviewed elsewhere, including another review in this special issue [63]. Co-infection with other STIs, including HIV, HSV, and Epstein–Barr virus (EBV), along with high diversity bacteriomes, are associated with decreased vaginal HPV clearance and persistent infection with HR-HPV (Figure 3), thus increasing the risk of HPV-related malignancies (Table 2) [64]. Cervical cancer is the most common AIDS-defining cancer in women, underscoring the interactions between HIV and HPV [62]. Co-infection with HPV and HSV also increases the risk of cervical squamous cell carcinoma or adenocarcinoma by 2- to 9-fold more than HPV infection alone [64]. Further, the presence of HPV may alter female genital tract eukaryotic virome diversity [65], suggesting a complex interaction between virome components.

NLD and BV-associated bacteriomes increased HPV persistence and carcinogenic progression (Table 2) [6,62,66,67]. Recent work has shown that the HPV E7 oncoprotein downregulates several host antimicrobial defense peptides that are normally secreted in response to bacterial LPS [67]. HPV colonization may, therefore, allow for the overgrowth of more pathogenic bacteria through the downregulation of host defense mechanisms. *Lactobacillus* spp., however, were immune to the antibacterial effects of these peptides, instead cleaving them for use as an amino acid source for growth [67]. *L. crispatus* and *L. gasseri* were also shown in vitro to downregulate the expression of HPV E6 and E7 oncoproteins [68], and therefore, these lactobacilli may play a direct role in preventing HPV virulence.

### 4.2. Bacterial Vaginosis

Bacterial vaginosis (BV) is the most common cause of abnormal vaginal discharge in reproductive-age women. This clinical disease occurs when disruption of the baseline vaginal bacteriome occurs, shifting away from a low-diversity LD bacteriome toward more diverse, NLD bacteriomes populated largely by anaerobes, including *Gardnerella vaginalis*, *Prevotella* spp., and *Fannyhessea vaginae* (previously known as *Atopobium vaginae*). Clinical symptoms manifest as vaginal malodor, discharge, itchiness, and vaginal inflammation [20]. More concerningly, BV is associated with numerous adverse health outcomes, including preterm labor and preterm birth, preterm rupture of membranes, chorioamnionitis, endometriosis [69], infertility [70], and an increased risk of STIs, including HR-HPV [62] and HIV [20,32]. Interestingly, smoking is a risk factor for BV, and prior work has shown that cigarette smoke chemicals are found in vaginal secretions and can induce lytic conversion of *Lactobacillus* bacteriophages [71], potentially explaining this association with BV.

Vaginal phageome communities differ between women with and without BV [5,16], with decreased *Lactobacillus phages* [16] and higher levels of bacillus and *Escherichia* phages [5]. Further, similar to vaginal bacterial community state types, vaginal bacteriophages form community groups dubbed viral state types (VSTs), which differ in diversity and are correlated with BV/LD status (Figure 3 and Table 2) [5,6]. Groups differ on the impact of BV/NLD on other eukaryotic viruses, such as anelloviruses, where some find an increased eukaryotic virome diversity in more diverse vaginal bacteriomes [6,8], while others found no difference [5]. This difference in phageomes indicates that phage populations may play a significant role in shaping vaginal bacterial communities and may represent a novel alternative to antibiotics for the treatment of BV.

### 4.3. Infertility and Preterm Birth

A few studies have examined the vaginal virome in IVF and infertility. A small study (n = 26) examining women undergoing in vitro fertilization (IVF) found a trend toward higher eukaryotic DNA virome diversity in women in whom IVF failed (did not achieve clinical pregnancy) [72]. Among women randomized to receive azithromycin antibiotic prophylaxis prior to embryo transfer, they found a higher diversity of herpesviruses and papillomaviruses [72]. However, the azithromycin group also received a different stimulation protocol due to significantly lower anti-Müllerian hormone (AMH) levels, a marker of ovarian reserve that correlates with IVF success rates. While, overall, there was no difference in pregnancy rates between prophylactic azithromycin administration groups, these protocol differences or underlying vaginal mucosal differences (as AMH tends to decrease as women approach menopause) may have impacted virome diversity, and this was not addressed. Another small study of 46 women showed a higher prevalence of a specific anellovirus, Torquetenovirus (TTV), in women with infertility, regardless of male or female factor infertility cause [10]. Anelloviruses are small circular DNA viruses that are often markers of immunosuppressed status but are not known to cause any disease and are often thought to be commensal viruses. These authors found no difference in *L. crispatus* dominance in the bacteriome in infertility, but individuals with TTV were less likely to have *L. crispatus*-dominant bacteriomes [10]. More research is needed in this area to better ascertain the impact the vaginal virome may play on infertility.

Prior older prospective studies in mice and humans have shown that increased vaginal eukaryotic DNA virome diversity increases the risk of preterm birth [33,73,74]. However, the phageome was not assessed in these studies. Since increased vaginal bacteriome diversity has also been strongly correlated with preterm birth [32], examination of the vaginal phageome would be of interest.

## 5. Vaginal Therapeutics

While antibiotic resistance and relapse are increasing, several promising microbially targeted or microbially derived products are in pre-clinical or clinical trials to improve women’s health.

### 5.1. Probiotics and Anti-Biofilm Agents

Treatment for BV has remained largely unchanged for almost half a century, with the first-line treatment being the antibiotic metronidazole [20,24]. While initial cure rates can approach 80%, more than half of women will experience recurrent BV within 6 months of therapy and up to 80% by one year [75,76]. This is partly due to the formation of biofilms by BV-associated bacteria, including *Gardnerella* and *Prevotella*, among others [77]. Biofilms provide a protective microenvironment for the growth of biofilm-forming organisms and are difficult to penetrate by antibiotics and host antimicrobial compounds. Ineffective penetration of antibiotics makes bacterial eradication difficult, allowing bacterial regrowth after the cessation of antibiotics. BV may also be re-introduced during sexual intercourse, and while it is not considered an STI, its prevalence is associated with sexual activity [20]. In vitro and in vivo studies have tested single-agent compounds on biofilm reduction. There was no overall efficacy found with antibiotics alone. However, promising results were noted with antiseptics, most notably a 4-log reduction in colony-forming units with iron sulfide in vitro, and octenidine in vivo, though resistance developed with repeated exposure to the latter. In vitro biofilm formation studies also noted promising results with the use of catatonic peptides, the synthetic phage endolysin enzyme PM-477, and plant extracts including *Thymbra capitata* essential oil, including in polymicrobial biofilms and ex vivo biofilms [77,78].

In a systematic review of treatment modalities for BV, both vaginally applied sucrose and probiotics resulted in higher clinical responses than metronidazole treatment [79]. These compounds show promise, especially if used in combination with standard of care or other novel agents to help prevent recurrence. A novel *Lactobacillus crispatus* probiotic (CTV-05) was studied in a randomized trial of weekly applications for 2 weeks after an initial metronidazole gel course, finding that colonization occurred in 61% of individuals at day 10, but waned once the applications were stopped [80]. Further supporting this, a recent systematic review of 11 clinical trials involving 1493 BV patients suggests lower recurrence rates at 3 months, and longer cure rates at 24 weeks in those given antibiotics followed by intravaginal probiotics versus those treated with antibiotics alone [81]. Another systematic review of 35 randomized controlled trials comprising 3751 subjects treated with probiotics also found a significant increase in BV cure rates and a significant reduction in BV recurrence rates [82].

Probiotics have also been trialed to help in the eradication of female genital tract HR-HPV infection and, thus, decrease progression to neoplasia. A placebo-controlled trial randomized 100 women with HR-HPV infection to receive either intravaginal *L. crispatus* strain chen-01 for 5 months or a placebo [83]. Six months after intravaginal *L. crispatus* chen-01 transplantation, women had significantly reduced HPV viral loads and decreased cytological transformation and vaginal inflammation, along with higher *L. crispatus* carriage and overall lower bacteriome diversity, similar to HPV-uninfected subjects [83]. These examples illustrate the need for further well-designed large-scale clinical trials to validate the impact of adjunctive intravaginal therapies to restore vaginal health.

### 5.2. Vaginal Microbiota Transplant

Another proposed therapeutic for recurrent BV is vaginal microbiota transplantation (VMT), which involves transplanting donor vaginal fluid rich in low diversity *L. crispatus*, *L. jensenii*, or *L. gasseri* (CSTs I, II, and V) transvaginally into recipients with BV or other vaginal disease states [84]. One study has demonstrated success with VMT, with four out of five recipients achieving remission of their BV during follow-up periods with no adverse effects. However, the participants received antibiotics for 2 years along with small amounts of fresh VMT, which also could have influenced the participants’ ability to achieve remission [85]. These studies offer additional insight into the connection between the vaginal ecosystem, the development of disease, and the possibility of preventing disease with the transplantation of vaginal microbial communities that are known to be optimal for vaginal health [84,85].

Although VMT may be a promising solution to BV, there are some drawbacks including the possibility of transferring pathogens, such as resistant fungi, or non-optimal vaginal communities [86,87]. Bacteriophages may be transplanted with donor VMT, which could result in transfer to undesirable genes including antimicrobial resistance (AMR), such as has been documented with fecal microbiota transfer (FMT) [88] and has led to bacteremia with fatal consequences [89]. While VMT would be less likely to result in bacteremia than FMT due to decreased mucosal surface area interaction, consideration should be given to similar screening protocols as with FMT. A prior study examining *Lactobacillaceae* genomes from commercial strains found that around half of the isolates harbored an integrated prophage, most often consisting of *Siphoviridae* or *Myoviridae* families, with at least four potential AMR genes encoded within the phage genomes [90]. This further highlights the importance of including AMR screening in VMT, which is not currently included in published screening protocols [84]. VMT may also be prohibitively expensive to lower income patients due to the procedure itself, and the need for donors to undergo rigorous screening to minimize adverse effects and ensure the safety of the recipients. Larger clinical trials are needed to evaluate the safety and efficacy of VMT for BV and its efficacy for other vaginal disease states.

### 5.3. Oleic Acid Treatment

Unsaturated long-chain fatty acids (uLCFAs) have shown antimicrobial activity against gram-positive organisms, and using a targeted approach, Zhu et al., 2024 investigated the impact of uLCFAs to modulate vaginal lactobacilli composition [21]. Oleic acid (OA) and similar long-chain fatty acids selectively inhibited *L. iners*, including metronidazole-resistant strains, while promoting the growth of non-*iners* lactobacilli [21]. The mechanism behind this was OA-induced upregulation of gene products in *L. crispatus*, *L. jensenii*, and *L. gasseri*, including a conserved putative fatty acid efflux pump and oleate hydrolase, that are missing from *L. iners* and BV-associated bacteria, making the latter susceptible to OA [21]. This study demonstrates the therapeutic potential of OA in the treatment of BV and preventing recurrence.

### 5.4. Phage Lysins

Phage lysins are bacteriophage lytic enzymes produced to cleave bacterial cell wall peptidoglycan for phage progeny release. They are effective for controlling bacterial populations on mucosal surfaces, rapidly lysing target gram-positive bacteria and resulting in bacterial death. They have been shown to act synergistically with antibiotics, and have been tested in a variety of diseases [91]. Group B streptococcus (GBS) often colonizes the urogenital tract and is the leading cause of neonatal meningitis. Women are screened prior to delivery and treated with antibiotics if vaginally colonized. Clinically pathogenic strains carry prophages whose genes enhance bacterial growth and biofilm formation [92], which may help GBS adapt to the vaginal environment and increase pathogenicity in neonates. However, a new therapeutic recombinant endolysin derived from a GBS prophage, EN534-C, was shown to lyse clinical GBS isolates but not beneficial vaginal lactobacilli in vitro [93]. This targeting of a specific bacterial pathogen is one example of the potential use of these novel antimicrobial agents in improving women’s health outcomes.

## 6. Conclusions and Future Research Directions

The complex interplay between the vaginal bacteriome, virome, and host immune defenses that impacts the development and persistence of female genital tract diseases is just beginning to be better understood, allowing for the development of novel, non-antibiotic antimicrobial compounds that show great promise in improving women’s health. However, much more research is needed. More research and standardization of virome protocols, from nucleic acid extraction to amplification and taxonomic identification pipelines, are needed to allow for cross-cohort comparisons to better differentiate the biological differences implicated with disease from procedural biases or population differences. Additionally, the implications of the virome in key areas of women’s health have yet to be fully investigated. Few studies have focused on the post-menopausal vaginal bacteriome and its impact on gynecological health [69], and the postmenopausal virome is completely unexplored. The RNA vaginal virome is also understudied. We know HIV and Zika virus are vaginally shed, which can have serious adverse effects on neonatal health [40,94,95], but the impact of other RNA viruses is unknown. Large-scale clinical trials are needed to test novel vaginal microbiota-based or targeted compounds to ensure safety and efficacy. A better understanding of the genetic bases and molecular mechanisms the virome uses to impact disease could lead to improved health outcomes in cervical cancer, preterm birth, infertility, BV, and HIV disease.

## Figures and Tables

**Table 1:** Vaginal virome manuscripts examining both the eukaryotic virome and bacteriophages.

Article	Sample Type/ Extraction Method	Microbiome	MGS Method & Depth	Country	N	Key Findings
Gosmann et al., 2017 [15]	Cervical swab VLP	DNA and RNA virome; bacteriome (16S)	Illumina MiSeq NR	South Africa	180	No distinct viral communities; HIV acquisition higher in NLD bacterial communities.
Jakobsen et al., 2020 [16]	Vaginal swab IVF VLP	DNA virome	Illumina NextSeq 45,000 PER/sample	Denmark	48	No difference in viral richness or alpha diversity by BV status, but virome beta diversity differed between BV+ and BV− subjects; BV negative samples contained ↑ abundance of lactobacilli phage.
de Costa et al., 2021 [17]	Vaginal swab Pregnant VLP	DNA and RNA virome	Illumina HiSeq NR	Brazil	107	No association between phage families and bacterial CST, preterm birth, or pregnancy outcome.
Zhang HT et al., 2021 [18]	Vaginal swab Pregnant VLP	DNA virome	Illumina Miseq pooled samples 220,000 reads/sample	China	60	↑ HPV subtype sequences in pregnant women with vaginitis. *Anelloviridae* and Noroviruses only found in pregnant women with vaginitis; *Microviridae* and *Herelleviridae* were absent from pregnant women without vaginitis.
Happel, A et al., 2021 [19]	Vaginal swab Adolescents Longitudinal WGS	DNA virome	Illumina NovaSeq 86 million/sample	South Africa	13	Prophages common esp in *L. crispatus* and were persistent; Number of CRISPR loci and prophages associated with microbiome stability.
Madere et al., 2022 [5]	Vaginal Swab +/− HIV VLP	DNA virome, bacteriome (16S)	Illumina NovaSeq 29 million PER/sample	South Africa	38	Vaginal phages cluster into communities that correlate with BV; ↑ Phage alpha diversity in BV; High proportion of lysogenic phage in BV.
Kaelin, E et al., 2022 [6]	CVL WGS, RCA	DNA virome, bacteriome (16S), HPV genotype	Illumina MiSeq 824,000 PER/sample	USA	23	↑ FRT inflammation yeilds ↓ virome richness and ↑ Anelloviridae; ↑ Lactobacillus phages found in LD; Phageome differs between LD and NLD; No association between HPV and inflammation.
Wang, J et al., 2022 [7]	Vaginal swab Postnatal mothers WGS	DNA virome	Illumina HiSeq NR	China	94	≤57% of neonatal virome derived from maternal vaginal viruses.
Li, Y et al., 2023 [8]	Vaginal swabs Cervical neoplasia vs. healthy VLP	Virome, bacteriome (16S)	Illumin NovaSeq 9.5 million PER/sample	China	161	Cervical cancer eukaryotic virome communities differed from neoplasia and healthy; No real evaluation of phages.
Britto, A et al., 2023 [9]	Cervical epithelial cells Healthy vs. HPV+ WGS, RCA	DNA Virome, bacteriome (16S)	MiSeq 1 mill PER (100 nt)/sample	Brazil	21	HPV infection associated with increased Type 1 IFN response; Only detected 2 viral families (*Anelloviridae*, *Papillomaviridae*), so virome analysis limited.
Da Costa, A et al., 2023 [10]	Vaginal swab Infertility VLP and RCA	Vaginal virome	Illumina NovaSeq	USA	46	Higher prevalence of TTV in women with infertility
Hugerth, LW et al., 2024 [11]	Vaginal swabs Healthy Longitudinal WGS	DNA virome, bacteriome	DNBSEQ-G400 NR	Denmark	49	Stable LD bacteriomes showed 1 log ↑ phage counts; Phage spikes noted with bacteriome shifts; ↑ phage reads during menstruation when bacteriome shifted during menstruation.
Li C et al., 2024 [12]	Vaginal swab WGS	DNA virome, bacteriome (MetaPhlAn3/ HUMAnN3)	Illumina Novaseq 5.8 Gbp/sample	Tibet and China	47	Sea-level vaginal samples show ↑ *Lactobacillus*; Viromes differ between altitude groups.
Huang L et al., 2024 [13]	Integrated prior vaginal MGS datasets (32 studies) WGS	DNA virome, Bacteriome, Fungeome	Various	Global	4472	Constructed the Vaginal Microbial Genome Collection, comprises >19,000 prokaryotic genomes, >14,000 viral OTUs, 38 fungal, 789 prokaryotes; 85.8% of vOTUs not found in other databases.
Kaelin, E et al., preprint [14]	CVL WLWH Longitudinal VLP, RCA	DNA virome, Bacteriome	Illumina NextSeq 10 million PER/sample	Peru	125	Bacteriome variation higher in HIV shedding; Virome diversity inversely related to ART duration.

IVF, in vitro fertilization; WGS, whole genome sequencing; VLP, virus-like particle; LD, *Lactobacillus*-dominant; NLD, non-*Lactobacillus* dominant; CST, community state type; CVL, cervicovaginal lavage; NR, not reported; PER, paired end reads; RCA, rolling circle amplification; WLWH, women living with HIV; ART, antiretroviral therapy; IFN, interferon; OTU, operational taxonomic unit; TTV, torquetenovirus; MGS, metagenomic sequencing; ↑ increased; ↓ decreased.

**Table 2:** Vaginal virome disease associations.

Vaginal Virus	Associated Disease/Risk
HR-HPV subtypes	↑ risk cervical cancer
Viral STI co-infections with HR-HPV	↑ risk of cervical cancer
Higher diversity bacteriomes with HR-HPV	↑ risk of cervical cancer
↑ Phageome diversity	BV
↑ Eukaryotic DNA viral diversity	↑ risk of preterm birth Trend ↑ IVF failure
↑ Torquetenoviruses	↑ Infertility

HR-HPV, human papillomavirus; STI, sexually transmitted infection; BV, bacterial vaginosis; IVF, in vitro fertilization; ↑ increased.

## Data Availability

Data sharing is not applicable.

## Abbreviations

The following abbreviations are used in this manuscript:

IVF	in vitro fertilization
CST	Community state type
WGS	whole genome sequencing
VLP	virus-like particle
LD	*Lactobacillus*-dominant
NLD	non-*Lactobacillus*-dominant
CVL	cervicovaginal lavage
WLWH	women living with HIV
ART	antiretroviral therapy
IFN	Interferon
OTU	operational taxonomic unit
TTV	Torquetenovirus
MGS	metagenomic sequencing
STI	sexually transmitted infections
TLR	toll-like receptor
AMH	anti-Müllerian hormone
VST	viral state types
VMT	vaginal microbiota transplantation
FMT	fecal microbiota transfer
uLCFAs	unsaturated long-chain fatty acids
OA	oleic acid
GBS	Group B streptococcus
